# The effect of omega-3 polyunsaturated fatty acid intake on blood levels of omega-3s in people with chronic atherosclerotic disease: a systematic review

**DOI:** 10.1093/nutrit/nuad020

**Published:** 2023-03-07

**Authors:** Nicole C Nayda, Jolene M Thomas, Christopher L Delaney, Michelle D Miller

**Affiliations:** are with the Caring Futures Institute, College of Nursing and Health Sciences, Flinders University, Bedford Park, South Australia, Australia; are with the Caring Futures Institute, College of Nursing and Health Sciences, Flinders University, Bedford Park, South Australia, Australia; is with the College of Medicine and Public Health, Flinders University, Bedford Park, South Australia, Australia; is with the Department of Vascular Surgery, Flinders Medical Centre, Bedford Park, South Australia, Australia; are with the Caring Futures Institute, College of Nursing and Health Sciences, Flinders University, Bedford Park, South Australia, Australia

**Keywords:** anti-inflammatory, antioxidant, atherosclerosis, cardiovascular disease, omega-3, supplementation

## Abstract

**Context:**

Atherosclerosis is a systemic pro-inflammatory and pro-oxidative disease, accounting for approximately a third of deaths globally. It has been proposed that omega-3s, through their antioxidant and anti-inflammatory properties, mitigate atherosclerotic disease progression. However, due to the systemic pro-inflammatory and pro-oxidative state of atherosclerosis, it is proposed that patients with atherosclerotic disease may have higher omega-3 requirements than the average requirement, due to increased nutrient utilization in anti-inflammatory and anti-oxidant processes.

**Objective:**

The aim of this review was to determine what dose and duration of omega-3 supplementation is required to reach a therapeutic blood level of omega-3s (eicosapentaenoic acid ≥150 µg/mL or omega-3 index ≥8%) in people with chronic atherosclerotic disease.

**Data Sources:**

This systematic review comprehensively searched MEDLINE, Emcare, Scopus, and CINAHL using key search terms for atherosclerotic disease, omega-3, supplementation, and blood levels of omega-3s.

**Data Extraction:**

Two reviewers independently screened 529 randomized controlled trials (RCTs) supplementing omega-3s in patients with chronic atherosclerotic disease.

**Data Analysis:**

In total, 25 journal articles from 17 original RCTs were included and assessed quantitatively. Supplementation at 1.8 g to 3.4 g per day for a 3-month–6-month duration, and at 4.4 g and above for as little as 1 month–6 months were identified as the most effective dosage ranges for increasing blood levels of omega-3s to therapeutic levels in people with atherosclerotic disease.

**Conclusions:**

Consideration should be given to routine omega-3 supplementation and to increasing the omega-3 dietary recommendations and upper limits of daily intake to improve clinical outcomes and reduce the risk of cardiac mortality in this population.

## INTRODUCTION

Atherosclerosis is a disease in which plaque hardens and narrows blood vessels, reducing the path for blood flow and the rate of oxygen delivery to organs and tissues.^1^ It is the underlying cause of many chronic cardiovascular diseases (CVDs), including coronary artery disease (CAD), peripheral artery disease (PAD), carotid artery disease, and renal artery disease, and these often coexist due to the systemic nature of the disease. Atherosclerotic disease is a major public health burden, with CAD and stroke accounting for 17.9 million (32%) deaths globally^2^ and CVD accounting for a global health expenditure of US$863 billion, expected to rise to US$1044 billion in 2030.^3^

Atherosclerosis is an immune-mediated condition associated with systemic inflammation and oxidation.^4^ Inflammation and oxidation are implicated in all stages of disease progression, including initiation, progression, destabilization, and plaque rupture.^4^^,^^5^ The initiation of atherosclerosis is by endothelial dysfunction, which promotes the recruitment and adhesion of blood monocytes, inflammatory white blood cells, to the vessel wall.^5^ Pro-inflammatory chemokines promote the entry of monocytes into the intima, where they mature to macrophages and take up modified low-density lipoprotein (LDL) particles to form foam cells.^5^ Adhesion molecules, cytokines, and growth factors are all inflammatory mediators that are implicated in these processes and contribute to the development of atherosclerotic plaque.^5^^,^^6^ Macrophages and T-cells within the atherosclerotic lesion also produce cytokines, eicosanoids, and reactive oxygen and nitrogen species, contributing to the chronic inflammatory burden of the condition.^6^ Destabilization and rupture of the plaque are also associated with the production of inflammatory cytokines.^7^ In addition, C-reactive protein is present in the vascular intima and contributes to atherogenesis by a number of mechanisms, including increasing LDL uptake into macrophages and promoting expression of adhesion molecules.^5^

With inflammation implicated in all stages of atherosclerosis, mitigating inflammation has the potential to slow or halt the progression of the condition.^8^ Omega-3 polyunsaturated fatty acids, namely alpha-linoleic acid (ALA), eicosapentaenoic acid (EPA) and docosahexaenoic acid (DHA), have demonstrated immune-modulating effects^9–11^ that have been proposed to attenuate atherosclerosis and decrease inflammation through multiple mechanisms.^12–14^ First, it is postulated that omega-3s are incorporated directly into atherosclerotic plaque, enhancing plaque stability, reducing macrophage infiltration, and preventing plaque rupture.^14^ The incorporation of omega-3s into cellular membranes also alters membrane fluidity, releasing endothelial relaxing factors, such as nitric oxide, and decreasing vascular tone.^12^ Furthermore, the enrichment of cells or tissues with omega-3s modulates the expression of pro-inflammatory adhesion proteins and cytokines.^13^ The metabolism of omega-3s also competes with the metabolism of omega-6s through the cyclooxygenase, lipoxygenase, and phospholipase A2 enzymes.^15^ This competition for metabolism was thought to be a rationale for omega-3’s anti-inflammatory effects by reducing the production of pro-inflammatory metabolites of omega-6s. However, recent evidence suggests that not all products of omega-6s are pro-inflammatory, and omega-6s also play a role in reducing inflammation and risk for coronary heart disease.^16^^,^^17^ This interaction between omega-3 and omega-6 metabolism in the context of inflammation is not fully understood, but it is thought that omega-3s and omega-6s likely work synergistically to produce anti-inflammatory effects.^16^^,^^17^

The systemic effects of omega-3s have been widely studied, with several meta-analyses and systematic reviews demonstrating the anti-hypertensive effect,^18–20^ and triglyceride-lowering effect of omega-3 supplementation.^21^^,^^22^ Prospective studies have demonstrated that consumption of omega-3s and higher blood levels of omega-3s are inversely related to cardiovascular mortality,^23^^,^^24^ and a 2019 meta-analysis of randomized controlled trials involving 127 477 participants has shown that marine omega-3 supplementation lowers risk for myocardial infarction, CAD death and total CAD compared with placebo.^25^ Despite this, there have been conflicting results from primary and secondary prevention trials (including VITAL,^26^ REDUCE-IT,^27^ and STRENGTH^28^) on the therapeutic benefits of omega-3s, which have precluded the development of an omega-3 reference range in the blood. Although there are no formal recommendations, an EPA blood level of 150 µg/mL or higher has been proposed as the most protective against major coronary events, based on a randomized clinical trial of 15 534 participants.^29^ This study found the risk of major coronary events was significantly decreased (20%) in participants with high (150 µg/mL or more) EPA concentrations compared with participants with low (less than 87 µg/mL) EPA concentrations.^29^ Additionally, an omega-3 index, a measurement of the percentage of EPA and DHA in erythrocyte cell membranes, of 8% or higher has been identified as being associated with the lowest risk of cardiac death.^30^^,^^31^ Evidence for this range is based on 6 primary and secondary prevention trials in which CVD outcomes and omega-3 indices were measured. In studies in which blood levels of EPA and DHA were given, results from a dose–response study were used to infer the omega-3 index.^31^ The study found the average omega-3 index associated with the lowest risk of cardiac death was approximately 8%.^31^ Following this, transformed data of a meta-analysis of 10 cohort studies comprising 27 469 participants further supported the proposed 8% omega-3 index as a target blood level.^30^ Although these are observational studies, and thus causation cannot be inferred, this is the closest to a reference range for an omega-3 index that we identified in the literature. Therefore, an EPA blood level of 150 µg/mL or higher and an omega-3 index of 8% or higher will be referred to as the therapeutic blood levels of omega-3s throughout this review. Given the systemic inflammation attributed to atherosclerosis, and the role of omega-3s in decreasing inflammation and their direct incorporation into cells, tissues, and atherosclerotic plaque, it is unknown whether higher doses or longer duration of supplementation is required to reach these therapeutic blood levels to account for the up-utilization of omega-3s in these processes.

Therefore, the aim of this review was to determine what dose and duration of omega-3 supplementation is required to reach a therapeutic blood level of omega-3s in people with chronic atherosclerotic disease.

## METHODS

This systematic review was registered on PROSPERO and followed the Preferred Reporting Items for Systematic Reviews and Meta-Analyses (PRISMA) reporting guidelines.

### Literature search

A comprehensive search was conducted using the electronic databases MEDLINE, Scopus, Emcare, and CINAHL on May 23, 2022. Key search terms included variations of omega-3, atherosclerosis, supplementation, and blood levels of omega-3 (see Table S1 in the Supporting Information online for the search strategies used across databases). Subject headings were used when available. The search was refined by age to adults (18 years and over), by language to English only, by study design to randomized controlled trials, and to human studies only. No restrictions were applied for publication date. Scoping searches were piloted prior to the confirmation of a search strategy to identify whether relevant studies existed and whether a similar review had already been published. Additionally, the reference lists of included articles were searched manually. Guidance regarding search terms and database selection was provided by a research librarian at our institution.

### Study selection

All search results were exported to EndNote 20 (Clarivate Analytics, London, United Kingdom) and imported into Covidence systematic review software (Veritas Health Innovation, Melbourne, Australia), and duplicates were removed. The PICOS criteria defining the inclusion and exclusion criteria for this review have been described in Table 1.

**Table 1:** PICOS criteria for inclusion and exclusion of studies

Parameter	Inclusion criterion	Exclusion criterion
**Participants**	Human studies with participants aged 18 years and over. Studies including participants with chronic atherosclerotic disease (including coronary artery disease, peripheral artery disease, or carotid artery disease)	Studies including participants with risk factors for cardiovascular disease but not established disease (eg, hyperlipidemia, hypertension, or diabetes) or studies where patients had established cardiovascular disease from non-atherosclerotic causes (eg, vasculitis). Studies where participants were pregnant or breastfeeding, due to the significant increase in basal metabolic rate and nutrient metabolism
**Interventions**	Studies administering omega-3 by supplementation or dietary intervention. Studies reporting the dose and duration of omega-3 supplementation	Studies administering mixed nutrient supplementation where no group received omega-3s alone. Studies incorporating dual treatments such as exercise with supplementation due to the increased physiological and nutritional demands. Studies where omega-3s were parenterally administered, as omega-3s would directly enter the bloodstream and affect omega-3 blood levels. Studies supplementing omega-3s post-surgical intervention only (eg, coronary bypass or valve replacement surgery), due to the increased inflammation and stress associated with surgery
**Comparisons**	Placebo or no intervention	
**Outcomes**	Studies reporting pre- and post-supplementation blood levels or percentage change in omega-3s in the blood	Studies not reporting pre- and post-supplementation blood levels or percentage change in omega-3s in the blood
**Study design**	Randomized controlled trials	Conference abstracts

Articles were screened independently in Covidence by 2 reviewers (N.C.N. and J.M.T.) against the inclusion and exclusion criteria. Studies were first screened by title and abstract and where studies appeared to meet the inclusion criteria, and for those where it was unclear, the full-text articles were obtained and again screened independently by the same 2 reviewers. Discrepancies between reviewers were resolved through verbal discussion.

### Data extraction

The author, year of publication, study design, type of CVD, sample size, male to female ratio, age, type and dose of intervention and placebo, baseline and post-intervention omega-3 levels, and the significance of findings were extracted from the included studies and tabulated. The extraction table was pilot tested for 1 study to ensure suitability before being confirmed for use. Dose was converted to grams per day, and where baseline and post-intervention omega-3 levels were not reported as a percentage, they were converted to µg/mL units to standardize the results. The percentage change in omega-3 levels was calculated by hand when not reported by the authors.

### Quality appraisal

A quality appraisal and risk-of-bias assessment was conducted on each included study using the “Academy of Nutrition and Dietetics Quality Criteria Checklist for Primary Research” tool (see Figure S1 in the Supporting Information online for the quality appraisal tool used).^32^ This is a validated tool chosen for its suitability for quantitative research. This tool assesses article quality by assigning “yes,” “no,” “unclear,” or “not applicable” to 4 relevance questions and 10 criteria, evaluating the presence of selection, attrition, performance, detection, and measurement bias, resulting in an overall positive, neutral, or negative strength paper.

## RESULTS

### Search results

**Figure 1:**
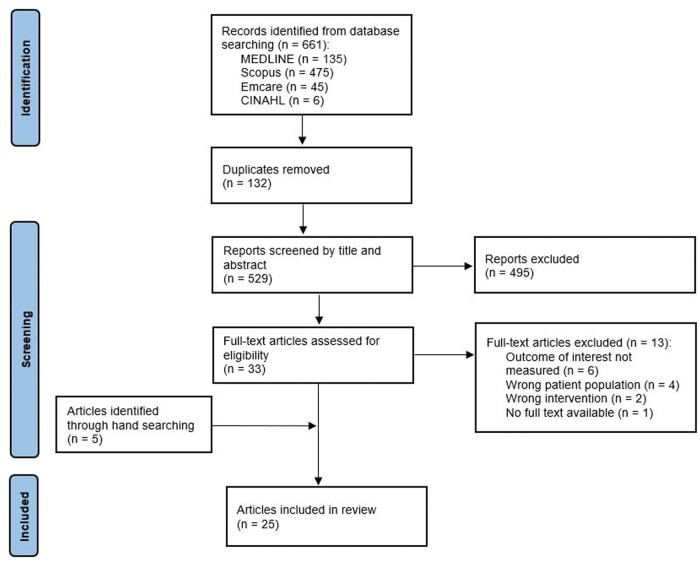
Flow diagram of the literature search process.

The search strategy yielded 661 results. Covidence identified and removed 132 duplicates, and the remaining 529 studies were screened by title and abstract. From this, the full text of 33 articles were assessed for eligibility. A total of 20 articles met the inclusion criteria. An additional 5 articles were identified from hand searching the reference lists of included articles. Of these 25 articles, 8 were identified as substudies,^33–40^ resulting in 21 unique samples of participants from 17 original studies. A flow diagram of the process of article selection is summarized in Figure 1.

### Study characteristics

A summary of the study characteristics can be found in Table 2.^41–57^ The date range of studies spanned 23 years, from 1999^54^ to 2022.^33^ CAD was investigated in 14 studies,^22^^,^^42^^,^^44–48^^,^^50–57^ PAD in 2 studies,^43^^,^^49^ and carotid artery disease in 1 study.^41^ Four studies investigating CAD^50^^,^^55–57^ and the 1 study investigating carotid artery disease^41^ supplemented patients prior to surgical intervention, while both studies in PAD supplemented patients with intermittent claudication, a lower severity of PAD.^58^ The sample size of participants ranged from 17^56^ to 1259,^42^ and the average age of participants ranged from 58.4^54^ to 74.6^45^ years. Most participants were male (64.8%^48^–100%^49^).

**Table 2:** Data summary table from studies investigating omega-3 supplementation and omega-3 blood levels in people with chronic atherosclerotic disease

Reference	Study design	Type of CVD	Sample size (% male)	Age of participants (years)	Intervention type and dosage (total daily dose)	Control	Duration	Baseline levels of omega-3s	Post-intervention levels of omega-3s (*P*-value)	Change in omega-3 levels (%)
Anderson et al (2014)^55^	SB RCT	CAD	Intervention: 12 (58) Control: 12 (75)	Intervention: 65.8 ± 9.9 Control: 63.1 ± 8.4	Capsules 3.4 g n3 PUFA daily (1.86 g EPA, 1.5 g DHA)	No treatment	17.5 d	Total FA (%) Intervention: EPA: .5 ± .1; DHA: 3.1 ± .7 Control: EPA: NR; DHA: NR	Total FA (%) Intervention: EPA: 1.7 ± .6 (*P *<* *.0001); DHA: 4.1 ± 2.0 (*P *=* *.19) Control: EPA: NR (NR) DHA: NR (NR)	Intervention: EPA: 240; DHA: 32.25 Control: EPA: NR; DHA: NR
Cawood et al (2010)^41^ Yusof et al (2013)^40^^,^^a^	DB RCT	Carotid artery disease	Intervention: 47 (68.1) Control: 53 (67.9)	Intervention: 72.0 ± 10.7 Control: 73.0 ± 8.3	1 capsule BD (.89 g EPA, .78 g DHA)	Olive oil capsule BD	Intervention: 22 (7–102)^b^ d Control: 21 (7–71)^b^ d	Plasma FA (%) Intervention: EPA: 1.3 ± .6; DHA: 3.7 ± 1.3 Control: EPA: 1.3 ± .6; DHA: 3.9 ± 1.2	Plasma FA (%) Intervention: EPA: 3.3 ± .9 (*P *<* *.001); DHA: 5.8 ± 1.2 (*P *<* *.001) Control: EPA: 1.3 ± .5 (NR); DHA: 4.2 ± 1.2 (NR)	Intervention: EPA: 153.8; DHA: 56.8 Control: EPA: .0; DHA: 7.7
Galan et al (2010)^42^	DB RCT	CAD	Intervention: 633 (79.2) Control: 626 (79.2)	Intervention: 60.41 (5.7–68.7)^b^ Control: 60.9 (54.5–68.1)^b^	2 capsules taken once daily (.4 g EPA, .2 g DHA)	Gelatine capsule	12 mo	Plasma FA (%) Intervention: EPA + DHA: 3.73 (2.92–4.91)^b^ Control: EPA + DHA: 4.04 (2.99–5.08)^b^	Plasma FA (%) Intervention: EPA + DHA: 5.34 (4.53–6.52)^b^ (NR) Control: EPA + DHA: 3.92 (2.99–4.81)^b^ (NR)	Intervention: EPA + DHA: 43.2 Control: EPA + DHA: –3.0
Garg et al (2006)^56^	RCT	CAD	Intervention: 9 (100) Control: 8 (88.9)	Intervention: 61.6 ± NR Control: 60.1 ± NR	6 × 1 g fish oil concentrate capsules daily (2.25 g EPA, 1.81 g DHA)	6 × 1 g olive oil capsules	6 wk	Plasma (µg/mL) Intervention: EPA: 8 ± 6; DHA: 13 ± 7 Control: EPA: 8 ± 6; DHA: 16 ± 8	Plasma (µg/mL) Intervention: EPA: 57 ± 29 (*P *<* *.01); DHA: 51 ± 22 (*P *<* *.01) Control: EPA: 12 ± 6 (NS); DHA: 20 ± 7 (NS)	Intervention: EPA: 612.5; DHA: 292.3 Control: EPA: 50.0; DHA: 25.0
Grenon et al (2013)^43^ Schaller et al (2017)^35^^,^^a^	DB RCT	PAD	Intervention: 40 (98) Control: 40 (98)	Intervention: 68 ± 7 Control: 69 ± 9	4 × capsules BD (2.6 g EPA, 1.8 g DHA)	Matched placebo	1 mo	Omega-3 Index (%) Intervention: 5.2 ± 1.7 Control: 4.6 ± 1.4	Omega-3 Index (%) Intervention: 9 ± 2% (*P *<* *.001) Control: NR (NS)	Intervention: 73.0 Control: 2.2
Heydari et al (2016)^44^	DB RCT	CAD	Intervention: 180 (82) Control: 178 (79)	Intervention: 60 ± 10 Control: 58 ± 10	4 × 1 g capsules (1.86 g EPA, 1.5 g DHA)	Corn oil	6 mo	Omega-3 Index (%) Intervention: 5.5 ± 1.8 Control: 5.7 ± 1.7	Omega-3 Index (%) Intervention: 9.96^c^ ± NR (*P *<* *.0001) Control: NR (NR)	Intervention: 81 Control: NR
Kalstad et al (2021)^45^ Mhyre et al (2022)^33^^,^^a^	DB RCT	CAD	Intervention: 438 (NR) Control: 443 (NR)	74.6 ± 3.6	3 capsules daily (.93 g EPA, .66 g DHA)	Matched corn oil placebo	24 mo	Serum FA (%) Intervention: EPA: 2.8 ± 1.4; DHA: 5.7 ± 1.4 Control: EPA: 2.9 ± 1.5; DHA: 5.7 ± 1.3	Serum FA (%) Intervention: EPA: NR (NR); DHA: NR (NR) Control: EPA: NR (NR); DHA: NR (NR)	Intervention: EPA: 87 (32, 165)^b^; DHA: 16 (2, 34)^b^ Control: EPA: –13 (–34, 20)^b^; DHA: –8 (–18, 6)^b^
Madsen et al (2007)^46^	DB RCT	CAD	41 (82.9)	63 ± 7	8 capsules daily (5.2 g PUFA; 4.3 g EPA and DHA)	8 capsules olive oil	12 wk	Platelet FA (%) Intervention: EPA: 1.49 ± .67; DHA: 2.78 ± .52 Control: EPA: 1.58 ± .50; DHA: 2.83 ± .71	Platelet FA (%) Intervention: EPA: 4.10 ± 1.03 (*P *<* *.01); DHA: 3.54 ± .45 (*P *<* *.01) Control: EPA: 1.46 ± .84 (NS); DHA: 2.78 ± .69 (NS)	Intervention: EPA: 175.2; DHA: 27.3 Control: EPA: –7.6; DHA: –1.8
Mazereeuw et al (2016)^47^	DB RCT	CAD	Intervention: 45 (76.1) Control: 47 (76.1)	Intervention: 63.8 ± 9.1 Control: 59.7 ± 7.9	3 capsules daily (1.2 g EPA, .6 g DHA)	3 capsules of soybean/corn oil at 1:1 ratio	12 wk	Plasma (µg/mL) Intervention: EPA: 26.7 ± 14.2; DHA: 47.3 ± 20.1 Control: EPA: 28.5 ± 16.6; DHA: 52.8 ± 23.8	Plasma (µg/mL) Intervention: EPA: 41.6 ± 34.4 (*P *<* *.01) DHA: 53.6 ± 29.3 (*P *<* *.01) Control: EPA: 23.4 ± 14.4 (NS); DHA: 45.9 ± 20.4 (NS)	Intervention: EPA: 55.8; DHA: 13.3 Control: EPA: –17.9; DHA: –11.7
Metcalf et al (2007)^57^	RCT	CAD	Intervention: 10 (80) Control: 10 (90)	Intervention: 63.5 ± 6.7 Control: 60.7 ± 14.5	10 mL fish oil concentrate daily (3 g EPA, 3 g DHA)	No treatment	33 (26–63)^b^ d	Plasma FA (%) Intervention: EPA: 1.17 ± .43; DHA: 3.58 ± 1.07 Control: EPA: 1.02 ± .41; DHA: 3.73 ± .61	Plasma FA (%) Intervention: EPA: 5.32 ± 1.32 (NR) DHA: 9.15 ± 1.39 (NR) Control: EPA: .98 ± .36 (NR); DHA: 3.57 ± .64 (NR)	Intervention: EPA: 354.7; DHA: 155.6 Control: EPA: –3.9; DHA: –4.3
Poreba et al (2017)^48^	DB RCT	CAD	Intervention: 36 (61.1) Control: 38 (68.4)	Intervention: 64.4 ± 6.7 Control: 66.7 ± 6.8	Drink (1 g EPA, 1 g DHA)	Matched placebo	3 mo	Serum (µg/mL) Intervention: EPA: 62.94 (45.17; 82.51)^b^ DHA: 283.51 (230.39; 343.82)^b^ Control: EPA: 60.73 (42.67; 80.92)^b^; HA: 289.57 (242.59; 327.43)^b^	Serum (µg/mL) Intervention: EPA: 164.42 (132.51; 203.77)^b^ (*P *<* *.0001); DHA: 437.91 (360.32; 512.80)^b^ (*P *<* *.0001) Control: EPA: 54.04 (36.73; 73.82)^b^ (NS); DHA: 270.97 (217.79; 362.07)^b^ (NS)	Intervention: EPA: 161.2; DHA: 54.5 Control: EPA: –11.0; DHA: –6.4
Ramirez et al (2019)^49^	DB RCT	PAD	Intervention: 11 (100) Control: 13 (100)	Intervention: 69 ± 8 Control: 73 ± 7	4 capsules daily (2.6 g EPA, 1.8 g DHA)	4 capsules soybean oil	3 mo	Omega-3 Index (%) Intervention: 5.5 ± 2.1 Control: 5.7 ± 1.5	Omega-3 Index (%) Intervention: 12.71^c^ ± NR (*P *<* *.001) Control: NR (NS)	Intervention: 130.9% Control: –7.0%
Saravanan et al (2010)^50^	DB RCT	CAD	Intervention: 52 (77) Control: 51 (82)	Intervention: 64 (58–71)^b^ Control: 68 (64–73)^b^	1 g capsule BD (.94 g EPA, .79 g DHA)	1 g capsule olive oil BD	Intervention: 16.5 (13–21)^b^ d Control: 17 (12–20)^b^ d	Serum FA (%) Intervention: EPA: 1.51 ± .98; DHA: 3.79 ± 1.37 Control: EPA: 1.76 ± 1.49; DHA: 3.82 ± 1.38	Serum FA (%) Intervention: EPA: 2.78 ± 1.13 (*P *<* *.001); DHA: 5.49 ± 1.02 (*P *<* *.001) Control: EPA: 1.53 ± .74 (NS); DHA: 4.19 ± 1.29 (NS)	Intervention: EPA: 84.1; DHA: 44.9 Control: EPA: –13.1; DHA: 9.7
Saravanan et al (2016)^34^^,^^a^	DB RCT	CAD	Intervention: 34 (79) Control: 27 (78)	Intervention: 64.5 (59–70)^b^ Control: 68 (64–73)^b^	1 g capsule BD (.94 g EPA, .79 g DHA)	1 g capsule olive oil BD	Intervention: 14 (9–35)^b^ d Control: 16.5 (7–34)^b^ d	Serum FA (%) Intervention: EPA: 1.38 ± .97; DHA: 3.83 ± 1.39 Control: EPA: 1.63 ± 1.18; DHA: 3.77 ± 1.38	Serum FA (%) Intervention: EPA: 2.48 ± 1.23 (*P *=* *.002); DHA: 5.59 ± 1.12 (*P *=* *.001) Control: EPA: 1.32 ± .84 (NS); DHA: 4.23 ± 1.26 (NS)	Intervention: EPA: 79.7; DHA: 46.0 Control: EPA: –19.0; DHA: 12.2
Sawada et al (2016)^51^	SB RCT	CAD	Intervention: 44 (81.5) Control: 43 (81.8)	Intervention: 67.8 ± 9.1 Control: 68.9 ± 8.8	2 × capsules daily (1.8 g EPA)	No treatment	6 mo	Plasma (µg/mL) Intervention: EPA: 69.3 ± 40.7; DHA: 158.9 ± 70.0 Control: EPA: 84.0 ± 50.7; DHA: 162.5 ± 64.6	Plasma (µg/mL) Intervention: EPA: 196.4 ± 51.4 (*P *<* *.0001) DHA: 134.2 ± 48.1 (*P *<* *.01) Control: EPA: 81.1 ± 45.0 (NS); DHA: 152.2 ± 57.7 (NS)	Intervention: EPA: 183.4; DHA: –15.5 Control: EPA: –3.5; DHA: –6.3
Seierstad et al (2005)^52^	DB RCT	CAD	Intervention: 20 (85) Control: 19 (94.7)	Intervention: 58 ± NR Control: 63 ± NR	700 g salmon fed 100% fish oil weekly (2.9 g marine n-3; 1:1.5 ratio)	700 g salmon fed 100% rapeseed oil weekly	6 wk	Serum (µg/mL) Intervention: EPA: 77 (77, 119)^b^; DHA: 145 (117, 202)^b^ Control: EPA: 56 (41, 80)^b^; DHA: 133 (86, 155)^b^	Serum (µg/mL) Intervention: EPA: 158 (83, 196)^b^ (*P *<* *.001); DHA: 185 (126, 239)^b^ (NS) Control: EPA: 52 (39, 68)^b^ (NS); DHA: 115 (80, 130)^b^ (*P *=* *.0444)	Intervention: EPA: 105.2; DHA: 27.6 Control: EPA: –7.1; DHA: –13.5
Tani et al (2017)^53^	RCT	CAD	Intervention: 53 (92) Control: 53 (83)	Intervention: 68 ± 11 Control: 66 ± 11	.9 g EPA capsule BD (1.8 g EPA)	No treatment	6 mo	Serum (µg/mL) Intervention: EPA: 59.8 (35.7, 97.3)^b^ Control: EPA: 60.1 (38.4, 91.9)^b^	Serum (µg/mL) Intervention: EPA: 162.7 (131.5, 200.7)^b^ (*P *< .0001) Control: EPA: 52.0 (36.5, 76.7)^b^ (NS)	Intervention: EPA: 172.1 Control: EPA: –13.5
Tani et al (2017)^38^^,^^a^ Tani et al (2019)^36^^,^^a^ Tani et al (2020)^37^^,^^a^	RCT	CAD	Intervention: 50 (92) Control: 50 (84)	Intervention: 67.5 ± 10.1 Control: 67.3 ± 10.4	.9 g EPA capsule BD (1.8 g EPA)	No treatment	6 mo	Serum (µg/mL) Intervention: EPA: 71 ± 45 Control: EPA: 70 ± 40	Serum (µg/mL) Intervention: EPA: 164 ± 54 (*P *<* *.0001) Control: EPA: 63 ± 36 (NS)	Intervention: EPA: 131.0 Control: EPA: –10.0
Tani et al (2020)^39^^,^^a^	RCT	CAD	Intervention: 30 (93.3) Control: 30 (83.3)	Intervention: 63.9 ± 10.6 Control: 65.5 ± 10.9	.9 g EPA capsule BD (1.8 g EPA)	No treatment	6 mo	Serum (µg/mL) Intervention: EPA: 72 ± 53 Control: EPA: 82 ± 45	Serum (µg/mL) Intervention: EPA: 163 ± 62 (*P *<* *.0001) Control: EPA: 66 ± 38 (NR)	Intervention: EPA: 126.4 Control: EPA: –19.5
Von Schacky et al (1999)^54^	DB RCT	CAD	Intervention: 111 (82) Control: 112 (78.6)	Intervention: 57.8 ± 9.7 Control: 58.9 ± 8.1	First 3 mo: 6 × 1 g capsules (2.12 g EPA, 1.29 g DHA) Next 21 mo: 3 × 1 g capsules (1.06 g EPA, .65 g DHA)	Matched placebo	24 mo	Erythrocyte FA (%) Intervention: EPA: .49 ± .2; DHA: 2.86 ± 1.1 Control: EPA: NR; DHA: NR	Erythrocyte FA (%) Intervention: EPA: 2.89 ± 1.0 (*P *<* *.05); DHA: 6.00 ± 1.2 (*P *<* *.05) Control: EPA: NR (NR); DHA: NR (NR)	Intervention: EPA: 489.8; DHA: 109.8 Control: EPA: NR; DHA: NR

All values are presented as mean ± SD unless otherwise specified.

a Substudy of another included article.

b Presented as median (range).

c Calculated from baseline levels and reported percentage increase.

*Abbreviations*: BD, twice daily; CAD, coronary artery disease; CVD, cardiovascular disease; DB, double blind; DHA, docosahexaenoic acid; EPA, eicosapentaenoic acid; FA, fatty acid; mo, months(s); NR, not reported; NS, not significant; PAD, peripheral artery disease; PUFA, polyunsaturated fatty acid; RCT, randomized controlled trial; SB, single blind; SD, standard deviation.

### Risk of bias

Each of the original 17 studies were assessed against the “Academy of Nutrition and Dietetics Quality Criteria Checklist for Primary Research” tool,^32^ and 14 papers received a positive rating^41–54^ and 3 received a neutral rating^55–57^ (see Table S2 in the Supporting Information online for the results of the quality appraisal). No studies received a negative rating. One study presented concerns for selection bias, with inclusion and exclusion criteria being poorly described.^56^ Five studies posed a risk for attrition bias, with methods of handling withdrawals being poorly described.^44^^,^^46^^,^^51^^,^^55^^,^^56^ Four studies did not report a sample size power calculation or did not meet the required sample size.^46^^,^^55–57^ A double blinded approach was used in 12 studies,^41–50^^,^^52^^,^^54^ while 2 studies were single blinded.^51^^,^^55^ In 3 studies, blinding, particularly of the outcome assessors, was poorly described, posing risk for detection bias.^53^^,^^56^^,^^57^ Matched placebos were utilized in the control group for 13 studies,^41–50^^,^^52^^,^^54^^,^^56^ while 4 did not administer a placebo,^51^^,^^53^^,^^55^^,^^57^ posing a risk for performance and detection bias. All studies used parallel controls.

### Type of omega-3 supplementation

Of the 17 studies, 14^41–47^^,^^49–51^^,^^53–56^ supplemented with capsules, and 1 study each used liquid fish oil concentrate,^57^ a fortified drink,^48^ and a dietary intervention with salmon.^52^ A combined omega-3 intervention, providing both EPA and DHA, was given in 15 studies,^41–50^^,^^52^^,^^54–57^ and EPA alone was used in 2 studies.^51^^,^^53^ No study administered exclusively DHA or ALA as an intervention. Seven studies supplemented with omega-3s as ethyl esters,^41^^,^^44^^,^^47^^,^^50^^,^^51^^,^^53^^,^^55^ 3 as triglycerides,^43^^,^^49^^,^^52^ and 1 as carboxylic acids,^45^ while 6 studies did not report the form of omega-3s provided.^42^^,^^46^^,^^48^^,^^54^^,^^56^^,^^57^

### Dose of omega-3 supplementation

The dose of combined omega-3 supplementation ranged from .6 g^42^ to 6.0 g^57^ total EPA and DHA per day. Both studies supplementing with only EPA provided a dose of 1.8 g EPA daily.^51^^,^^53^ For combined omega-3 supplementation, EPA was provided in a higher dose than DHA in all but 2 studies, 1 of which provided an EPA:DHA ratio of 1:1.1^52^, and the other provided equal amounts of EPA and DHA.^48^ Of the remaining combined supplementation studies, the dose ratio of EPA:DHA ranged from 1.14:1^41^ to 2:1,^42^^,^^47^ with 1 study not reporting separate EPA and DHA content, only the total amount.^46^

### Duration of omega-3 supplementation

The duration of omega-3 supplementation ranged from 16.5 days^50^ to 24 months.^45^^,^^54^

### Method of measurement of omega-3 blood levels

The method of measuring omega-3 levels differed across studies. Measurements in plasma (as µg/mL),^47^^,^^51^^,^^56^ serum (as µg/mL),^48^^,^^52^^,^^53^ and percentage of plasma fatty acids^41^^,^^42^^,^^57^ were used in 3 studies each. Two studies measured omega-3 levels as percentage of serum fatty acids,^45^^,^^50^ while erythrocyte fatty acids,^54^ percentage of platelet fatty acids,^46^ and percentage of total fatty acids in whole blood^55^ were used in 1 study each. Omega-3 index was used in 3 studies.^43^^,^^44^^,^^49^

### Change in omega-3 blood levels

Omega-3 supplementation increased blood levels of omega-3s in all 17 studies. Of these, omega-3 blood levels were significantly increased from baseline in 11 studies (*P < *.05),^41^^,^^43^^,^^44^^,^^46–50^^,^^53^^,^^54^^,^^56^ while 3 studies did not report statistical significance.^42^^,^^45^^,^^57^ Two studies saw a significant increase in EPA (105%–240%, *P *<* *.001) and a non-significant increase in DHA (27.6%–32.3%, *P *>* *.05) in the intervention group.^52^^,^^55^ One study supplementing with EPA only saw a significant increase in EPA (183%, *P *<* *.0001) and a significant decrease in DHA (–15.5%, *P *<* *.01).^51^

The increase in omega-3 levels of EPA and DHA observed in plasma (µg/mL) in combined supplementation studies ranged from 55.8% (*P *<* *.01)^47^ to 612.5% (*P *<* *.01)^56^ and from 13.3% (*P *<* *.01)^47^ to 292.3% (*P *<* *.01),^56^ respectively.

In serum (µg/mL), change in EPA and DHA levels ranged from 105.2% (*P *<* *.001)^52^ to 172.1% (*P *<* *.0001)^53^ and 27.6% (*P *>* *.05)^52^ to 54.5% (*P *<* *.0001),^48^ respectively.

When looking at studies measuring omega-3s as the percentage of plasma fatty acids, increases in levels of EPA and DHA ranged from 153.8% (*P *<* *.001)^41^ to 354.7% (*P*-value not reported)^57^ and 56.8% (*P *<* *.001)^41^ to 155.6% (*P-*value not reported), respectively.^57^

In studies measuring omega-3s as the percentage of serum fatty acids, change in EPA and DHA ranged from 79.7% (*P *<* *.001)^50^ to 87% (*P*-value not reported)^45^ and 16% (*P*-value not reported)^45^ to 46.0% (*P *<* *.001), respectively.^50^

Increases in omega-3 index ranged from 73.0% (*P *<* *.001)^43^ to 130.9% (*P *<* *.001).^59^

In the control groups, 7 studies reported decreases in EPA that ranged from –3.5% (*P* > .05) to –19.0% (*P*-value not reported),^45–48^^,^^50–53^^,^^57^ 6 reported nonsignificant decreases in DHA ranging from –1.8% to –11.7% (*P *>* *.05),^45–48^^,^^51^^,^^57^ 1 reported a significant decrease in DHA (–13.5%, *P *<* *.044),^52^ 1 reported a nonsignificant decrease in omega-3 index (–7.0%, *P *>* *.05),^49^ and 1 reported a decrease in combined EPA and DHA content in the control group at study completion when compared with baseline (–2.97%, *P*-value not reported).^42^

In the studies where blood levels were measured as either omega-3 index or quantified to µg/mL, all studies reported baseline omega-3 levels that were below the therapeutic blood levels. After supplementation, 7 of the 17 studies^43^^,^^44^^,^^48^^,^^49^^,^^51–53^ reached therapeutic levels of omega-3s.

### Impact of supplementation duration and dose on omega-3 blood levels

Both studies supplementing with only EPA^51^^,^^53^ used the same dose (1.8 g) and duration of supplementation (6 months) and resulted in similar increases in blood levels of EPA (172.1%, *P* < .000153 and 183.4%, *P* < .000151).

At similar baseline levels of plasma fatty acids (EPA+DHA: 3.73 [2.92–4.91]%^42^, EPA 1.17 ± .43%, DHA: 3.58 ± 1.07%^57^), a higher dose (6 g) of combined omega-3 supplementation at shorter duration (33 [26–63] d) saw greater increases in plasma fatty acid percentage of omega-3s (EPA: 534.7%, DHA: 155.6%, *P-*value not reported)^57^ than a lower dose (.6 g) of combined omega-3 supplementation for longer duration (12 months) (combined EPA and DHA: 43.16%, *P*-value not reported).^42^ Similarly, this was observed in studies using the omega-3 index, where supplementation at 4.4 g per day for 1 month^43^ and 3 months^49^ saw a similar (73%, *P *<* *.001) and greater (131%, *P *<* *.01) increase in blood levels than supplementation at 3.4 g per day for 6 months (81%, *P *<* *.0001)^44^ where baseline omega-3 indexes were comparable (5.2 ± 1.7%^43^, 5.5 ± 2.1%^49^).

Similarly, in combined supplementation studies measuring blood levels in plasma (µg/mL), higher dose supplementation of 4.06 g for the shorter duration of 6 weeks^56^ saw greater increases in blood levels (EPA: 612.5%, DHA: 292.3%, *P *<* *.01) than lower dose supplementation of 1.8 g for the longer duration of 12 weeks (EPA: 55.8%, DHA: 13.3%, *P *<* *.01)^47^. However, baseline levels of EPA and DHA differed, with the lower baseline study (EPA: 8 ± 6 µg/mL, DHA: 13 ± 7 µg/mL)^56^ finding a greater increase in blood levels after supplementation compared with the study that had a higher baseline level (EPA: 26.7 ± 14.2 µg/mL, DHA: 47.3 ± 20.1 µg/mL).^47^

### Optimal dose and duration of omega-3 supplementation to reach therapeutic blood levels

The 7 studies^43^^,^^44^^,^^48^^,^^49^^,^^51–53^ that saw increases in blood levels to the therapeutic range were studies involving supplementing with doses of omega-3s of ≥1.8 g daily. From doses of 1.8 g–3.4 g of omega-3s, daily supplementation for 3 months–6 months was sufficient to reach therapeutic blood levels. In 1 study using dietary intervention with daily salmon equating to an omega-3 intake of 2.9 g per day, therapeutic levels were achieved in 6 weeks.^52^ However, at higher doses of 4.4 g of omega-3s daily, supplementation for as little as 1 month was sufficient to increase blood levels to therapeutic ranges.^43^ Two studies supplementing with doses of above 1.8 g did not see increases into the therapeutic range; however, in these studies, baseline levels measured in plasma (µg/mL) (EPA: 26.7 ± 14.2, DHA: 47.3 ± 20.1^47^, EPA: 8 ± 6, DHA: 13 ± 7^56^) were much lower than the other study using this biomarker (EPA: 69.3 ± 40.7, DHA: 158.9 ± 70.0).^47^^,^^56^

### Impact of the type of cardiovascular disease on omega-3 blood levels

All studies saw increases in omega-3 blood levels, regardless of the type of CVD studied. Both studies investigating PAD^43^^,^^49^ measured blood levels by omega-3 index, with the baseline omega-3 index of PAD participants ranging from 4.6 ± 1.4% to 5.5 ± 2.1%. Both studies supplemented with the same combined dose of 4.4 g EPA and DHA per day with a similar baseline omega-3 index in both intervention groups (5.2 ± 1.7% and 5.5 ± 2.1%); however, the duration of supplementation differed at 1 month^43^ and 3 months.^49^ Supplementation for 3 months saw an increase of 131% in the omega-3 index (*P *<* *.001), compared with only a 73% increase after 1 month (*P *<* *.001); however, both reached therapeutic levels at study completion. Only 1 study investigating CAD used the omega-3 index, and baseline levels ranged from 5.5 ± 1.8% to 5.7 ± 1.7%,^44^ which was higher compared with studies measuring the omega-3 index and investigating PAD.^44^ In this study, CAD participants were supplemented with a combined dose of 3.36 g EPA and DHA for 6 months and saw an increase of 81% in the omega-3 index (*P *<* *.0001), reaching therapeutic levels at study completion. The 1 study in carotid artery disease^41^ measured blood levels in plasma fatty acids and revealed a baseline EPA and DHA of 1.3 µg/mL and 3.8 µg/mL, respectively.

## DISCUSSION

This novel review explored the effect of omega-3 supplementation on blood levels of omega-3s in people with chronic atherosclerotic disease. The review aimed to determine the dose and duration of omega-3 supplementation required to achieve a therapeutic blood level of omega-3s (EPA blood level greater than 150 µg/mL^29^ and an omega-3 index of 8%^30^^,^^31^) in people with chronic atherosclerotic disease. As it is proposed that the pro-inflammatory and pro-oxidative state of atherosclerosis may increase the utilization of omega-3s in anti-inflammatory and anti-oxidant processes, it is important to determine an optimal dose and duration of supplementation to reduce the risk for major coronary events and sudden cardiac death.

### Baseline levels of omega-3s

At baseline, all participants were below the therapeutic blood levels of omega-3s, with most studies reporting baseline omega-3 values closer to half of this. Lower baseline levels of omega-3s were identified in studies investigating PAD when compared with studies investigating CAD. This is proposed to be secondary to PAD exerting a greater pro-inflammatory and pro-oxidative response compared with CAD.^60^^,^^61^ Additionally, as both studies investigating PAD only included patients with intermittent claudication, a less severe form of PAD, this effect may be further pronounced in patients with critical limb ischemia, the most severe form of PAD, with an increased inflammatory and oxidative burden. However, as a limitation of case–control research and due to the limited number of studies investigating PAD, the exact cause of this difference is unable to be established and could be related to several other factors, including poorer dietary intake and increased comorbidities among the PAD participants. When comparing baseline levels of omega-3s in PAD participants with those in studies conducted in the healthy population, omega-3 levels were consistently higher in the healthy population.^59^^,^^62^^,^^63^ This is consistent with published literature concluding that people with chronic atherosclerotic disease have lower baseline levels of omega-3s when compared with the healthy population.^59^ Given that omega-3s are proposed to reduce the progression of atherosclerosis, support optimal mental health and functioning,^64^ and promote wound healing,^65^ consideration of making omega-3 supplementation routine practice in patients with chronic atherosclerotic disease is justified.

### Post-intervention levels of omega-3s

All omega-3 supplementation, both dietary interventions and supplementation with capsules, saw increases in blood levels of omega-3s regardless of the dosage and duration of supplementation. This indicates that although atherosclerosis is pro-inflammatory and pro-oxidative, and hence these patients may have higher requirements due to increased nutrient utilization, omega-3 incorporation into the blood still occurs with supplementation. The extent of increases in omega-3 blood levels varied across studies and suggests an element of individual variation in response to supplementation, which is consistent with the relevant literature.^29^ A review by Superko et al^29^ proposes that genetic differences in fatty acid metabolism, body weight, age, gender, physical activity, and baseline levels of omega-3s all contribute to the variability in individual blood level response to supplementation. There is also evidence to suggest that the carboxylic acid and triglyceride forms of omega-3s are more easily absorbed in the body than ethyl esters.^66^^,^^67^ Given that the form of omega-3 supplementation varied, with some studies not reporting this information,^42^^,^^46^^,^^48^^,^^54^^,^^56^^,^^57^ this may account for some variation in blood levels between studies. It is also important to consider the variation in time taken for omega-3s to incorporate into different biomarkers. Serum and plasma omega-3 levels, including individual fatty acid levels as well as triglyceride and phospholipid fatty acid levels, increase in a matter of hours to days after omega-3 consumption.^68^ However, erythrocyte membranes reflect longer-term omega-3 intake aggregated over 120 days, the half-life of erythrocytes.^68^ This indicates that omega-3 levels measured in erythrocytes, including the omega-3 index, are less acutely affected by short-term supplementation, especially durations of less than 120 days. Despite this, only 2 studies^43^^,^^49^ measuring omega-3 index supplemented for durations of less than 120 days, at 1 month and 3 months, and both studies reached therapeutic levels. This indicates these short supplementation durations were sufficient to see changes in blood levels. Hence, the variation in time taken for omega-3 incorporation into different biomarkers does not affect the findings in this review.

Greater increases in omega-3 blood levels were generally observed in studies where baseline levels of omega-3s were lower, when dose and duration of supplementation was similar. This is consistent with findings from other randomized controlled trials supplementing with omega-3s.^69–71^ When looking at studies supplementing with omega-3s in the healthy population, increases in blood levels of omega-3s were greater in the healthy population at similar doses and duration.^62^^,^^72^ In 1 study, supplementation at 1.68 g for 6 months saw increases in the omega-3 index of 161%–171% in the healthy population.^62^ This increase is double what was observed in CAD participants being supplemented with higher doses (3.36 g) for the same duration, even when baseline levels of omega-3s were higher in the study in the healthy population.^44^ Similarly, a study supplementing healthy participants with .6 g combined EPA and DHA for only 4 weeks saw the same increase in plasma fatty acids (42.2%)^72^ at 4 weeks as CAD participants did at 12 months (43.2%), when baseline levels were comparable.^42^ This indicates that there is a difference between the healthy population and people with atherosclerotic disease in either omega-3 incorporation into the blood or omega-3 utilization, likely due to the systemic inflammatory and oxidative state of atherosclerosis.

### Effect of omega-3 type, duration, and dosage on post-intervention omega-3 levels

This review provides evidence that doses of 1.8 g to 3.4 g of omega-3s per day for 3-months–6-months duration, and supplementation at 4.4 g and above for as little as 1 month–6 months is the most effective at reaching therapeutic levels of omega-3s. All doses used across studies were higher than what is recommended by the National Health and Medical Research Council (NHMRC) in Australia, the American Heart Association (AHA), and the National Health Service in England. The NHMRC suggest the adequate intake of total long-chain omega-3s for healthy adults aged 19 years and over, comprising EPA, DHA, and docosapentaenoic acid (DPA), is .16 g/day and .09 g/day for men and women, respectively.^73^ Additionally, the NHMRC have published the upper limit of omega-3 intake for adults as 3 g per day, which is the highest daily intake likely to not cause toxicity or other negative effects.^73^ The National Institute of Health in the United States have no published adequate intake for EPA and DHA; however, the AHA recommend at least 2 servings of fish per week to provide an average of 250 mg EPA plus DHA,^74^ and the National Health Service in England and the British Dietetic Association recommend 1 serve of oily fish per week to provide an average of 450 mg of EPA plus DHA per day.^75^^,^^76^ The AHA additionally recommend an increased omega-3 intake in patients with established CAD of 1 g of EPA plus DHA per day.^77^ The United States Food and Drug Administration initially also recommended an upper limit of 3 g combined EPA and DHA per day but have recently found that supplementation with up to 5 g per day is safe when used as recommended.^78^ These upper limits are based on some evidence that suggests high levels of omega-3 fatty acids can impair the immune response and prolong bleeding time.^73^

Eight studies supplemented with doses higher than the NHMRCs upper limit,^43^^,^^44^^,^^46^^,^^49^^,^^54–57^ with 1 doubling this dose.^57^ Only 1 of these studies reported adverse effects, with 4 participants reporting mild gastrointestinal discomfort—similar to their control group, which reported 3 cases.^54^ Two of these studies supplemented patients prior to cardiac surgery, and both reported no significant differences in bleeding or postsurgical blood loss in the intervention group compared with controls.^44^^,^^57^ This suggests that omega-3 supplementation above the NHMRC’s upper limit of dietary intake may be safe in this patient population and not result in bleeding complications. This is supported by a multicenter trial that demonstrated no relationship between the omega-3 index and bleeding in a large cohort of acute myocardial infarction patients,^79^ and is also supported by the FDA’s recent increase in upper limit recommendations.^78^ Two studies supplemented with doses above the FDA’s higher upper limit of 5 g, both of which also reported no side effects.^46^^,^^57^

As greater increases in omega-3 levels have been observed in the healthy population with the same dose and duration of supplementation, this supports the hypothesis that the pro-inflammatory and pro-oxidative state of atherosclerosis increases nutrient utilization. Due to this fact, and that no significant adverse effects were reported with supplementation above upper limits, it is possible that people with atherosclerosis require a higher upper limit of omega-3s than the healthy population. Additionally, increases in omega-3 blood levels to therapeutic levels did not occur until 1.8 g of total EPA and DHA day, which is greater than the AHA’s recommendation of 1 g per day in patients with established CAD. This also suggests that patients with chronic atherosclerotic disease require higher recommended daily intakes of omega-3s to reach therapeutic levels.

As no studies supplemented with ALA or DHA alone, and the majority of studies supplemented with a combined EPA and DHA intervention, the most effective type of omega-3 supplementation and optimal ratio of EPA to DHA supplementation could not be established within the scope of this review. As there is conflicting evidence for the most effective type of omega-3 supplementation and the ideal ratio of EPA to DHA, further trials investigating different types of omega-3 supplementation would be beneficial to inform supplementation recommendations.

### Quality assessment

The quality assessment, as per the “Academy of Nutrition and Dietetics Quality Criteria Checklist for Primary Research” tool,^32^ indicates that some studies are at risk of bias. This is due to poorly described processes of handling withdrawals,^44^^,^^46^^,^^51^^,^^55^^,^^56^ some studies not reporting a sample size power calculation or not meeting the required sample size,^46^^,^^55–57^ some studies not providing information on the blinding of researchers,^53^^,^^56^^,^^57^ and some studies not blinding participants by the omission of a placebo.^51^^,^^55^ However, all studies used objective measurements at an appropriate level of precision and used appropriate statistical analyses, therefore being at a low risk for measurement bias. With all studies being randomized controlled trials, and nearly all being classified as positive strength papers (only 3 being classified as neutral strength papers), the level of evidence provided in this literature review is strong.

### Strengths and limitations

A strength of this review is the systematic approach followed to select relevant articles, and the use of a validated critical appraisal tool^32^ to assess the level of evidence and risk of bias. The inclusion of only randomized controlled trials, at high levels of evidence, is another strength of this review, as this is recommended for providing evidence on the effectiveness of healthcare interventions.^80^ The lack of literature in the PAD and carotid artery disease populations, equating to only 3 out of 17 included studies in this review, makes it difficult to compare the effect of the type of CVD on blood levels of omega-3s, both pre- and post-supplementation. This may also limit the applicability of the determined optimal dose and duration of supplementation in these populations. Furthermore, despite the samples of participants being comparable across studies in terms of age, male to female ratio, and baseline characteristics, there was a much higher prevalence of males across studies, with 1 study exclusively recruiting males.^49^ Although statistics suggest CVD is more common in males,^81^^,^^82^ a sample largely consisting of male participants introduces bias and may limit the applicability of the findings to females. Finally, as assessing clinical outcomes associated with omega-3 supplementation was outside the scope of this review, the findings cannot directly be translated into improved clinical outcomes. However, the published literature has demonstrated improvements in clinical and cardiovascular outcomes, including reduced cardiovascular mortality, with increased omega-3 blood levels.^83^^,^^84^

## CONCLUSION

This systematic review provides evidence that, although atherosclerosis is pro-inflammatory and pro-oxidative, and hence patients may have higher requirements secondary to increased nutrient utilization, supplementation with omega-3s still increased blood levels. As all studies reported baseline omega-3 levels below therapeutic ranges, the concept of routine supplementation in patients with atherosclerotic disease should be explored. Higher dose supplementation was identified as the most effective at increasing blood levels of omega-3s, even when provided for shorter durations. Omega-3 supplementation of between 1.8 g and 3.4 g per day for 3-months–6-months duration, and supplementation at 4.4 g and above for as little as 1-month–6 months was identified as the most effective strategy for increasing blood levels of omega-3s to therapeutic levels. Further research comparing blood levels of omega-3s between people with chronic atherosclerotic disease and the healthy population would be beneficial to establish the degree to which atherosclerosis compromises omega-3 blood levels. Additionally, research focusing on the optimal type and dose ratio of EPA to DHA would be beneficial. This research could then inform the recommended omega-3 doses for chronic atherosclerotic diseases, and perhaps a revised upper limit for this population, which could result in a clinically significant improvement in outcomes for CVD patients by achieving therapeutic blood reference ranges, thus reducing the likelihood of major coronary events.

## Supplementary Material

nuad020_Supplementary_Data

## Supporting Information

The following Supporting Information is available through the online version of this article at the publisher’s website.

*Table S1* **Search strategies used across databases**

*Table S2* **Results of the quality appraisal**

*Figure S1* **Academy of Nutrition and Dietetics Quality Criteria Checklist for Primary Research tool**
