# Supplementary material for: The effect of omega-3 polyunsaturated fatty acid intake on blood levels of omega-3s in people with chronic atherosclerotic disease: a systematic review
Source: Nutr Rev. 2023 Mar 7;81(11):1447–61. doi: 10.1093/nutrit/nuad020 (PMC10563859; doi:10.1093/nutrit/nuad020)
Supplement: nuad020_Supplementary_Data [file nuad020_supplementary_data.zip › nuad020_Supplementary_Data/Supporting Materials Appendix S1 Search strategy used across databases.docx]

Appendix S1: Search strategy used across databases.

| **MEDLINE** | |
| --- | --- |
| **Concept** | **Search Terms** |
| 1  (title/ abstract) | "Omega-3*" or "n-3" or "alpha-linolenic acid" or "ALA" or "eicosapentaenoic acid" or "EPA" or "docosahexaenoic acid" or "DHA" or "fish oil" |
|  | Subject Headings: ‘Fatty Acids, Omega-3,’ ‘Eicosapentaenoic Acids,’ ‘Docosahexaenoic Acids’ |
| 2 | "atherosclero*" or "peripheral arterial disease" or "peripheral artery disease" or "PAD" or "coronary artery disease" or "coronary heart disease" or "CAD" or "CHD" OR "carotid artery disease" OR "arteriosclerosis" OR "ischaemic heart disease" OR "ischemic heart disease" OR "IHD" OR "heart disease*" OR "cardiovascular disease*" OR "CVD" OR "ischaemi" OR "ischemi*"OR “coronary” |
|  | Subject Headings: ‘Atherosclerosis,’ ‘Arteriosclerosis,’ ‘Coronary Artery Disease,’ ‘Peripheral Arterial Disease,’ ‘Carotid Artery Diseases’ |
| 3 | "supplement*" or "ingest*" or "consum*" or "intake*" or "administration" |
|  | Subject Heading: ‘Dietary Supplements’ |
| 4 | "Blood levels" or "serum levels" or "plasma levels" or "blood omega-3*" or "serum omega-3*" or "plasma omega-3*" or "omega-3 level*" or "omega-3 concentration" or "blood n-3" or "serum n-3" or "plasma n-3" or "n-3 level*" or "n-3 concentration" or "serum alpha-linolenic acid" or "plasma alpha-linolenic acid" or "alpha-linolenic acid level*" or "alpha-linolenic acid concentration" or "serum ALA" or "plasma ALA" or "ALA level*" or "ALA concentration" or "serum eicosapentaenoic acid" or "plasma eicosapentaenoic acid" or "eicosapentaenoic acid level*" or "eicosapentaenoic acid concentration" or "serum EPA" or "plasma EPA" or "EPA level*" or "EPA concentration" or "serum docosahexaenoic acid" or "plasma docosahexaenoic acid" or "docosahexaenoic acid level*" or "docosahexaenoic acid concentration" or "serum DHA" or "plasma DHA" or "DHA level*" or "DHA concentration" |

Concept 1, 2, 3, and 4 joined with ‘AND.’

Limited to: All adult studies, Human, Randomised control trials.

Returned 135 articles.

| **Emcare** | |
| --- | --- |
| **Concept** | **Search Terms** |
| 1  (title/ abstract) | "Omega-3*" or "n-3" or "alpha-linolenic acid" or "ALA" or "eicosapentaenoic acid" or "EPA" or "docosahexaenoic acid" or "DHA" or "fish oil" |
|  | Subject Headings: ‘Fatty Acids, Omega-3,’ ‘Eicosapentaenoic Acids,’ ‘Docosahexaenoic Acids’ |
| 2 | "atherosclero*" or "peripheral arterial disease" or "peripheral artery disease" or "PAD" or "coronary artery disease" or "coronary heart disease" or "CAD" or "CHD" OR "carotid artery disease" OR "arteriosclerosis" OR "ischaemic heart disease" OR "ischemic heart disease" OR "IHD" OR "heart disease*" OR "cardiovascular disease*" OR "CVD" OR "ischaemi" OR "ischemi*"OR “coronary” |
|  | Subject Headings: ‘Atherosclerosis,’ ‘Arteriosclerosis,’ ‘Coronary Artery Disease,’ ‘Peripheral Arterial Disease,’ ‘Carotid Artery Diseases’ |
| 3 | "supplement*" or "ingest*" or "consum*" or "intake*" or "administration" |
|  | Subject Heading: ‘Dietary Supplements’ |
| 4 | "Blood levels" or "serum levels" or "plasma levels" or "blood omega-3*" or "serum omega-3*" or "plasma omega-3*" or "omega-3 level*" or "omega-3 concentration" or "blood n-3" or "serum n-3" or "plasma n-3" or "n-3 level*" or "n-3 concentration" or "serum alpha-linolenic acid" or "plasma alpha-linolenic acid" or "alpha-linolenic acid level*" or "alpha-linolenic acid concentration" or "serum ALA" or "plasma ALA" or "ALA level*" or "ALA concentration" or "serum eicosapentaenoic acid" or "plasma eicosapentaenoic acid" or "eicosapentaenoic acid level*" or "eicosapentaenoic acid concentration" or "serum EPA" or "plasma EPA" or "EPA level*" or "EPA concentration" or "serum docosahexaenoic acid" or "plasma docosahexaenoic acid" or "docosahexaenoic acid level*" or "docosahexaenoic acid concentration" or "serum DHA" or "plasma DHA" or "DHA level*" or "DHA concentration" |

Concept 1, 2, 3, and 4 joined with ‘AND.’

Limited to: All adult studies, Human, Randomised control trials

Returned 45 articles.

| **Scopus** | |
| --- | --- |
| **Concept** | **Search Terms** |
| 1 (title/ abstract) | "Omega-3*" or "n-3" or "alpha-linolenic acid" or "ALA" or "eicosapentaenoic acid" or "EPA" or "docosahexaenoic acid" or "DHA" or "fish oil" |
|  | "atherosclero*" or "peripheral arterial disease" or "peripheral artery disease" or "PAD" or "coronary artery disease" or "coronary heart disease" or "CAD" or "CHD" OR "carotid artery disease" OR "arteriosclerosis" OR "ischaemic heart disease" OR "ischemic heart disease" OR "IHD" OR "heart disease*" OR "cardiovascular disease*" OR "CVD" OR "ischaemi" OR "ischemi*" OR "coronary" |
| 2 | Supplement* or ingest* or consum* or intake* or administration |
| 3 | "Blood levels" or "serum levels" or "plasma levels" or "blood omega-3*" or "serum omega-3*" or "plasma omega-3*" or "omega-3 level*" or "omega-3 concentration" or "blood n-3" or "serum n-3" or "plasma n-3" or "n-3 level*" or "n-3 concentration" or "serum alpha-linolenic acid" or "plasma alpha-linolenic acid" or "alpha-linolenic acid level*" or "alpha-linolenic acid concentration" or "serum ALA" or "plasma ALA" or "ALA level*" or "ALA concentration" or "serum eicosapentaenoic acid" or "plasma eicosapentaenoic acid" or "eicosapentaenoic acid level*" or "eicosapentaenoic acid concentration" or "serum EPA" or "plasma EPA" or "EPA level*" or "EPA concentration" or "serum docosahexaenoic acid" or "plasma docosahexaenoic acid" or "docosahexaenoic acid level*" or "docosahexaenoic acid concentration" or "serum DHA" or "plasma DHA" or "DHA level*" or "DHA concentration" |
| 4 | “Randomi?ed controlled trial" or "randomi?ed control trial" or "RCT" |

Concept 1, 2, 3, and 4 joined with ‘AND.’

Limit to journal article and article.

Returned 475 articles.

| **CINAHL** | |
| --- | --- |
| **Concept** | **Search Terms** |
| 1 (title/ abstract) | "Omega-3*" or "n-3" or "alpha-linolenic acid" or "ALA" or "eicosapentaenoic acid" or "EPA" or "docosahexaenoic acid" or "DHA" or "fish oil" |
|  | Subject Headings: ‘Fatty Acids, Omega-3’, ‘Eicosapentaenoic Acids,’ or ‘Docosahexaenoic Acids’ |
| 2 | Supplement* or ingest* or consum* or intake* or administration |
|  | Subject Headings: ‘Dietary Supplements’ or ‘Dietary Supplementation’ |
| 3 | "Blood levels" or "serum levels" or "plasma levels" or "blood omega-3*" or "serum omega-3*" or "plasma omega-3*" or "omega-3 level*" or "omega-3 concentration" or "blood n-3" or "serum n-3" or "plasma n-3" or "n-3 level*" or "n-3 concentration" or "serum alpha-linolenic acid" or "plasma alpha-linolenic acid" or "alpha-linolenic acid level*" or "alpha-linolenic acid concentration" or "serum ALA" or "plasma ALA" or "ALA level*" or "ALA concentration" or "serum eicosapentaenoic acid" or "plasma eicosapentaenoic acid" or "eicosapentaenoic acid level*" or "eicosapentaenoic acid concentration" or "serum EPA" or "plasma EPA" or "EPA level*" or "EPA concentration" or "serum docosahexaenoic acid" or "plasma docosahexaenoic acid" or "docosahexaenoic acid level*" or "docosahexaenoic acid concentration" or "serum DHA" or "plasma DHA" or "DHA level*" or "DHA concentration" |
| 4 | “Randomi#ed controlled trial" or "randomi#ed control trial" or "RCT" |

Concept 1, 2, 3, and 4 joined with ‘AND.’

Returned 6 articles.
