# Supplementary material for: The effect of omega-3 polyunsaturated fatty acid intake on blood levels of omega-3s in people with chronic atherosclerotic disease: a systematic review
Source: Nutr Rev. 2023 Mar 7;81(11):1447–61. doi: 10.1093/nutrit/nuad020 (PMC10563859; doi:10.1093/nutrit/nuad020)

Appendix S2: The Academy of Nutrition and Dietetics Quality Criteria Checklist for Primary Research Tool.


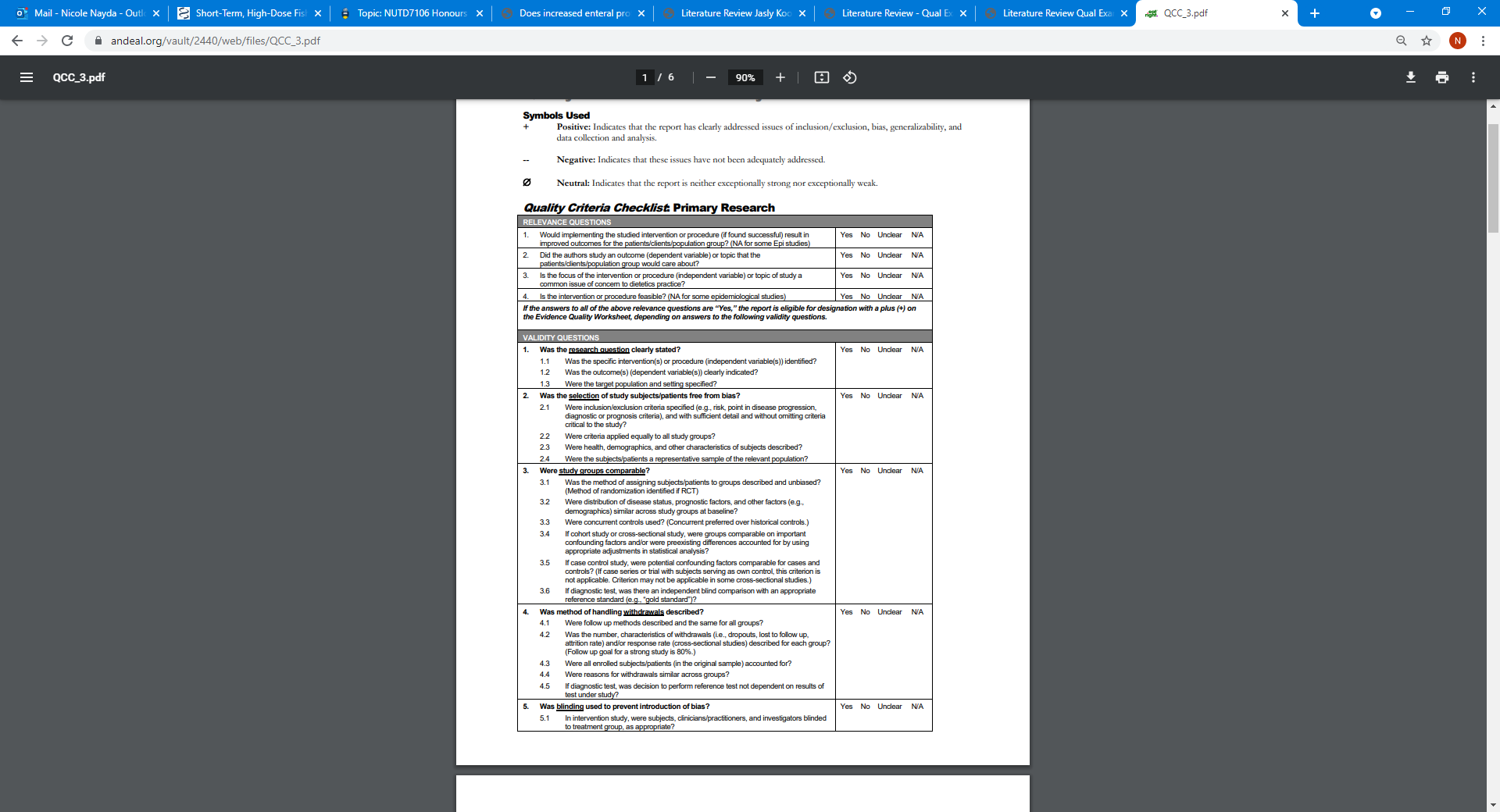


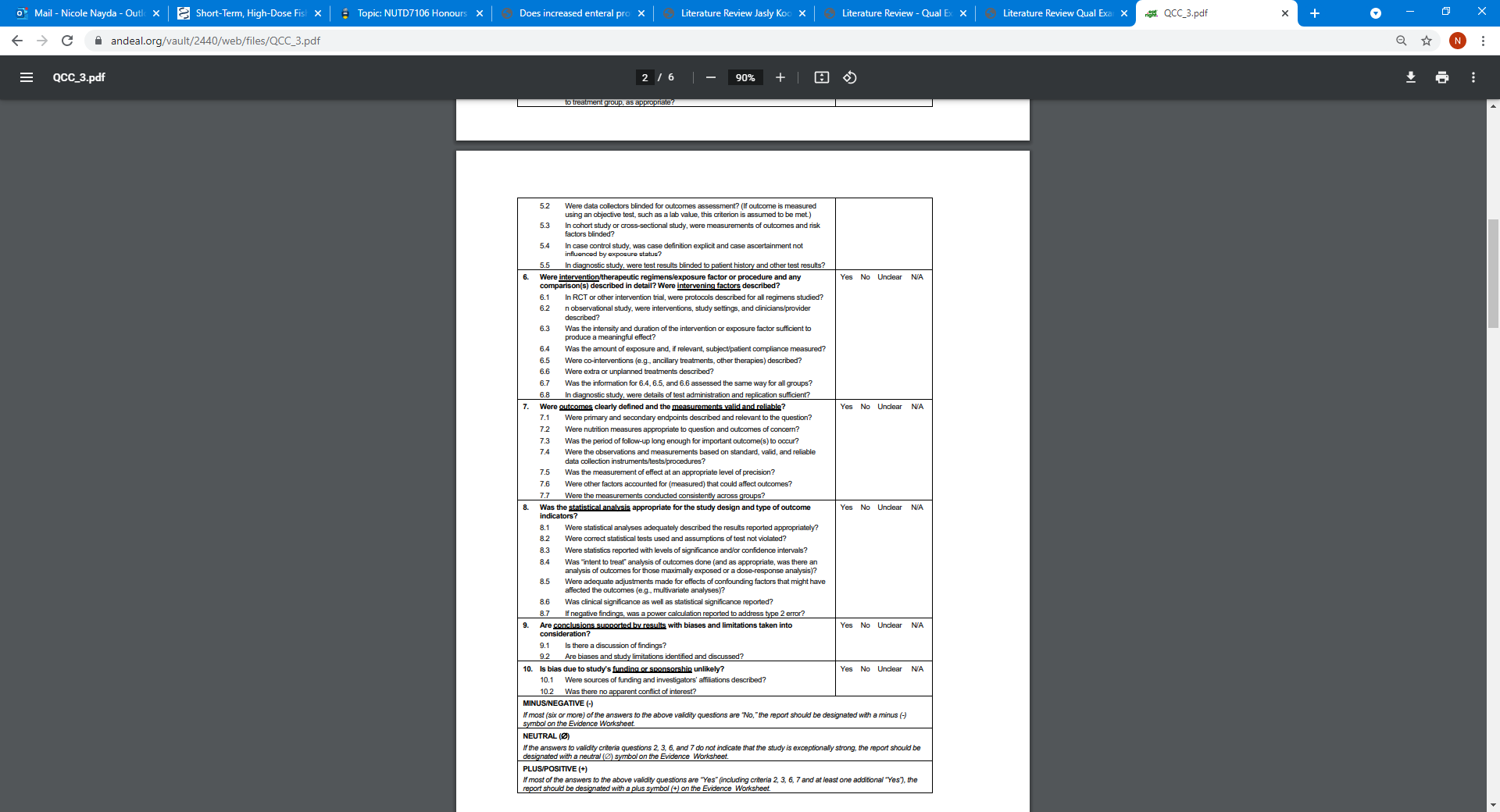

Supplement: nuad020_Supplementary_Data [file nuad020_supplementary_data.zip › nuad020_Supplementary_Data/Supporting Materials Appendix S2 Quality Appraisal Tool.docx]
