# Supplementary material for: The effect of omega-3 polyunsaturated fatty acid intake on blood levels of omega-3s in people with chronic atherosclerotic disease: a systematic review
Source: Nutr Rev. 2023 Mar 7;81(11):1447–61. doi: 10.1093/nutrit/nuad020 (PMC10563859; doi:10.1093/nutrit/nuad020)
Supplement: nuad020_Supplementary_Data [file nuad020_supplementary_data.zip › nuad020_Supplementary_Data/Supporting Materials Appendix S3 Results of the quality appraisal.docx]

Appendix S2: Results of the quality appraisal using the Academy of Nutrition and Dietetics Quality Criteria Checklist for Primary Research.

| **RELEVANCE QUESTIONS** | Anderson et al. 2014 | | Cawood et al. 2010 | | Galan et al. 2010 | | Garg et al. 2006 | | Grenon et al. 2013 | | Heydari et al. 2016 | | Kalstad et al. 2021 | | Madsen et al. 2007 | Mazereeuw et al. 2016 | Metcalf et al. 2007 |
| --- | --- | --- | --- | --- | --- | --- | --- | --- | --- | --- | --- | --- | --- | --- | --- | --- | --- |
| 1. Would implementing the studies intervention or procedure (if found successful) result in improved outcomes for the patients/clients/population group? | Y | | Y | | Y | | Y | | Y | | Y | | Y | | Y | Y | Y |
| 2. Did the authors study an outcome (dependent variable) or topic that the patients / clients/ population group would care about? | Y | | Y | | Y | | Y | | Y | | Y | | Y | | Y | Y | Y |
| 3. Is the focus of the topic of study (independent variable) a common issue of concern to dietetics practice? | Y | | Y | | Y | | Y | | Y | | Y | | Y | | Y | Y | Y |
| 4. Is the intervention or procedure feasible? | Y | | Y | | Y | | Y | | Y | | Y | | Y | | Y | Y | Y |
| **VALIDITY QUESTIONS** |  | |  | |  | |  | |  | |  | |  | |  |  |  |
| 1. Was the research question clearly stated? | Y | | Y | | Y | | Y | | Y | | Y | | Y | | Y | Y | Y |
| 2. Was the selection of study subjects/patients free from bias? | Y | | Y | | Y | | UC | | Y | | Y | | Y | | Y | Y | Y |
| 3. Were study groups comparable? | Y | | Y | | Y | | Y | | Y | | Y | | Y | | Y | Y | UC |
| 4. Was method of handling withdrawals described? | N | | Y | | Y | | N | | Y | | UC | | Y | | UC | Y | Y |
| 5. Was blinding used to prevent introduction of bias? | N | | Y | | Y | | UC | | Y | | Y | | Y | | Y | Y | Y |
| 6. Were exposure factor and any comparison(s) described in detail? Were the intervening factors described? | N | | Y | | Y | | Y | | Y | | Y | | Y | | Y | Y | Y |
| 7. Were outcomes clearly defined and the measurements valid and reliable? | Y | | Y | | Y | | Y | | Y | | Y | | Y | | Y | Y | Y |
| 8. Was the statistical analysis appropriate for the study design and type of outcome indicators? | UC | | Y | | Y | | UC | | Y | | Y | | Y | | UC | Y | UC |
| 9. Are conclusions supported by results with biases and limitations taken into consideration? | Y | | Y | | Y | | Y | | Y | | Y | | Y | | Y | Y | Y |
| 10. Is bias due to study’s funding or sponsorship unlikely? | Y | | Y | | Y | | Y | | Y | | Y | | Y | | Y | Y | Y |
| **Overall Quality Rating** | Ø | | + | | + | | Ø | | + | | + | | + | | + | + | Ø |
| **RELEVANCE QUESTIONS** | | Poreba et al. 2017 | | Ramirez et al. 2019 | | Saravanan et al 2010 | | Sawada et al. 2016 | Seierstad et al. 2005 | Tani et al. 2017 | | Von Schacky et al. 1999 | |  |  |  |  |
| 1. Would implementing the studies intervention or procedure (if found successful) result in improved outcomes for the patients/clients/population group? | | Y | | Y | | Y | | Y | Y | Y | | Y | |  |  |  |  |
| 2. Did the authors study an outcome (dependent variable) or topic that the patients / clients/ population group would care about? | | Y | | Y | | Y | | Y | Y | Y | | Y | |  |  |  |  |
| 3. Is the focus of the topic of study (independent variable) a common issue of concern to dietetics practice? | | Y | | Y | | Y | | Y | Y | Y | | Y | |  |  |  |  |
| 4. Is the intervention or procedure feasible? | | Y | | Y | | Y | | Y | Y | Y | | Y | |  |  |  |  |
| **VALIDITY QUESTIONS** | |  | |  | |  | |  |  |  | |  | |  |  |  |  |
| 1. Was the research question clearly stated? | | Y | | Y | | Y | | Y | Y | Y | | Y | |  |  |  |  |
| 2. Was the selection of study subjects/patients free from bias? | | Y | | Y | | Y | | Y | Y | Y | | Y | |  |  |  |  |
| 3. Were study groups comparable? | | Y | | Y | | Y | | Y | Y | Y | | Y | |  |  |  |  |
| 4. Was method of handling withdrawals described? | | Y | | Y | | Y | | N | NA | Y | | Y | |  |  |  |  |
| 5. Was blinding used to prevent introduction of bias? | | Y | | Y | | Y | | N | Y | N | | Y | |  |  |  |  |
| 6. Were exposure factor and any comparison(s) described in detail? Were the intervening factors described? | | Y | | Y | | Y | | Y | Y | Y | | Y | |  |  |  |  |
| 7. Were outcomes clearly defined and the measurements valid and reliable? | | Y | | Y | | Y | | Y | Y | Y | | Y | |  |  |  |  |
| 8. Was the statistical analysis appropriate for the study design and type of outcome indicators? | | Y | | Y | | Y | | Y | Y | Y | | Y | |  |  |  |  |
| 9. Are conclusions supported by results with biases and limitations taken into consideration? | | Y | | Y | | Y | | Y | Y | Y | | Y | |  |  |  |  |
| 10. Is bias due to study’s funding or sponsorship unlikely? | | Y | | Y | | Y | | Y | Y | Y | | Y | |  |  |  |  |
| **Overall Quality Rating** | | + | | + | | + | | + | + | + | | + | |  |  |  |  |

Y = yes; N = no; UC = unclear; NA = not applicable; + = positive rating; - = negative rating; Ø = neutral rating
